# Metabolomics and Plant Defense

**DOI:** 10.3390/metabo15030171

**Published:** 2025-03-03

**Authors:** Junxing Lu, Shitou Xia

**Affiliations:** 1 Chongqing Key Laboratory of Plant Environmental Adaptations, College of Life Science, Chongqing Normal University, Chongqing 401331, China; 2 Hunan Provincial Key Laboratory of Phytohormones and Growth Development, College of Bioscience and Biotechnology, Hunan Agricultural University, Changsha 410128, China

## 1. Introduction

Plant metabolomics is pivotal in understanding plant defense mechanisms against environmental stresses [1,2], which enables researchers to profile the complex metabolic changes in plants in response to biotic and abiotic factors such as pathogens, drought, and temperature extremes [3,4]. By analyzing metabolic fingerprints, scientists can identify key metabolites that are upregulated or synthesized during stresses, including defense phytohormones like salicylic acid, jasmonic acid, and ethylene, which are crucial for mounting an effective resistance [5,6]. Additionally, metabolomic studies reveal the accumulation of secondary metabolites such as flavonoids and phenolics, which often possess antioxidant properties and can protect plants from oxidative damage caused by various stresses [7]. These are fundamental for understanding plant biology and developing strategies to enhance crop resilience and improve agricultural sustainability in the face of climate change.

The field of metabolomics has become instrumental in deciphering the intricate metabolic responses of plants to biotic and abiotic stresses, particularly in the context of disease resistance and environmental adaptability [8]. This Special Issue of *Metabolites*, entitled “Metabolomics and Plant Defense” contains nine original research articles and one review, focusing on distinct plant–pathogen interactions and plant–environmental stress responses to enhance our understanding of how plants reprogram their metabolism as a defense strategy against pathogens and environmental challenges.

## 2. Metabolomics Insights into Plant Pathogen Resistance

In recent years, metabolomics has emerged as a powerful tool for understanding the complex metabolic responses of plants to biotic stresses, particularly in the context of disease resistance [1,6]. This Special Issue features four original research articles on distinct plant–pathogen interactions, which collectively contribute to our understanding of how plants employ metabolic reprogramming in resistance against pathogens.

Singh et al. (contribution 1) investigated the metabolic responses of the wild tomato species *Solanum cheesmaniae* to the pathogen *Alternaria solani*, which causes early blight. Untargeted metabolomics was applied to identify significant metabolic biomarkers and pathways associated with plant resistance. The study identified 10,943 metabolite features with 3371 compounds annotated using the KEGG database, implicating secondary metabolites, cofactors, steroids, terpenoids, and fatty acids. A total of 541 upregulated and 485 downregulated metabolite features were identified, which play roles in defense, infection prevention, signaling, plant growth, and homeostasis under stress. Orthogonal partial least squares discriminant analysis (OPLS-DA) revealed 34 upregulated and 41 downregulated biomarker metabolites, which could contribute to disease-resistant metabolic traits and tomato breeding programs.

Barley (*Hordeum vulgare*), a highly versatile cereal crop, is widely cultivated but is susceptible to fungal pathogens, notably the ascomycete *Pyrenophora teres f. teres* (*Ptt*), which causes the ‘net blotch net form’ (NBNF) disease. Hamany Djande et al. (contribution 2) provided insights into the metabolomic reconfiguration of barley plants primed with 3,5-dichloroanthranilic acid (3,5-DCAA) in response to *Ptt* infection, shedding light on how priming can arm barley with a rapid and robust defense mechanism against necrotrophic fungi, a group known to cause significant crop losses worldwide. Both untargeted and targeted metabolomics analyses were conducted, revealing a complex interplay of metabolites that are reprogrammed in primed barley. Key metabolites such as 5-oxo-proline and citric acid were significantly associated with the priming response. These findings advance our understanding of the regulated and reprogrammed metabolic responses that constitute defense priming in barley against *Ptt*.

The tea industry, a vital sector in global agriculture, faces significant challenges from diseases including gray blight caused by *Pestalotiopsis*-like species. Zheng et al. (contribution 3) provided valuable insights into the metabolic defense mechanisms of tea plants (*Camellia sinensis*) in response to this disease. Using a multi-omics approach, the researchers identified 64 differentially accumulated metabolites, including increased levels of antimicrobial compounds like caffeine and (−)-epigallocatechin 3-gallate, along with a decrease in the synthesis of (+)-catechin and (−)-epicatechin. A dynamic modulation of different branches of the flavonoid biosynthetic pathway was revealed in tea plants under fungal challenge. The findings underscore the strategic reprogramming of tea plant metabolism, prioritizing the production of potent antimicrobial flavanols while reducing the allocation of resources to less bioactive compounds. The metabolic shift is likely crucial for bolstering the plant’s defense against fungal pathogens.

Diseases such as Moko, caused by *Ralstonia solanacearum,* also pose significant threats to the global banana industry. Mihai et al. (contribution 4) examined the metabolic response of *Musa* spp. from the Ecuadorian coast to this disease, shedding light on their defense mechanisms. A higher accumulation of secondary metabolites with antioxidant capacity was identified in diseased samples compared to healthy ones, suggesting strategic metabolic reprogramming in response to pathogenic attack. The study employed a multi-omics approach, utilizing LC-MS to analyze active compounds with antioxidant properties in four common *Musa* varieties. The results indicated an upregulation of phenolic compounds and flavonoids such as kaempferol and quercetin glycosides, which are crucial for the plant’s defense against *R. solanacearum*.

These four studies collectively illustrate the dynamic and complex nature of plant metabolic responses to pathogen infection and reveal how plants employ a suite of metabolic changes, including the upregulation of secondary metabolites and antioxidants, to combat pathogen invasion. These metabolic shifts not only provide immediate defense but also prime the plant for future encounters, highlighting the plant’s ability to “remember” and respond more effectively to subsequent attacks. Metabolomic approaches offer a window into the plant’s chemical arsenal, allowing scientists to identify and manipulate key metabolic pathways to enhance disease resistance.

## 3. Metabolomics Insights into Plant Environmental Stress Adaptation

The intricate interplay between environmental stresses and plant metabolism is akin to a complex dance, where each step, choreographed by nature, carries profound implications for agriculture and food security. Within this Special Issue, four original research articles delve into the metabolic responses of plants under various stressors.

Hamany Djande et al. (contribution 5) explored the metabolic reprogramming in barley plants following the application of dichlorinated functional analogs of salicylic acid, shedding light on how these plants bolster their defenses against environmental stresses. Employing a multi-omics approach, the research revealed significant alterations in both primary and secondary metabolites, with a particular emphasis on the phenylpropanoid pathway—a known marker of induced resistance in plants. The application of 3,5-dichloroanthranilic acid, 2,6-dichloropyridine-4-carboxylic acid, and 3,5-dichlorosalicylic acid to barley at the third leaf stage triggered a metabolic response, underscoring the potential of these dichlorinated compounds as inducers of acquired resistance. The study observed the accumulation of barley-specific metabolites, for instance hordatines and precursors, indicating a strategic metabolic reprogramming that enhances the plant’s defense mechanisms.

In the face of environmental adversities, β-cyclocitral (βCC) emerges as a beacon of hope for bolstering the resilience of *Solanum lycopersicum* (tomato). This apocarotenoid, an oxidative product of β-carotene, primes plants for stress acclimation without impeding growth—a delicate balance rarely achieved. Deshpande and Mitra (contribution 6) meticulously documented how βCC induces metabolic changes, enhancing both defense mechanisms and growth in tomato plants. Through a sophisticated LC-MS/MS analysis, the research identified 57 compounds. Among which, amino acids and phytophenols, upregulated by βCC, not only fortify the plant against biotic stresses but also support its growth. This dual action underscores the wisdom of plant biology, where βCC orchestrates a temporal separation in metabolite accumulation, optimizing resource allocation. The findings are a testament to βCC’s promise as a metabolic signal, priming crops for a fortified defense against environmental challenges.

Amid the growing challenges posed by acidic soils that hinder global crop production, pyroligneous acid (PA) emerges as a promising ally for tomato plants. Aluminum toxicity, a pervasive issue in such soils, can be mitigated by PA application, according to a recent study by Ofoe et al. (contribution 7). This research uncovers the metabolic nuances of how PA treats tomato plants under aluminum stress, revealing a complex interplay of central carbon metabolism (CCM). Forty-eight differentially expressed metabolites were identified as involved in CCM, indicating PA’s role in regulating plant metabolism to adapt to aluminum stress. Notably, PA increased metabolites associated with glycolysis and the tricarboxylic acid (TCA) cycle, suggesting a shift towards enhanced energy production and organic acid biosynthesis, which is crucial for aluminum tolerance.

Understanding plant responses to water stress is critical for agricultural resilience, particularly in light of climate change. Abbey et al. (contribution 8) explored the metabolic shifts in Mexican mint (*Plectranthus amboinicus*) under varying watering regimes. The research reveals that 68 key metabolites of central carbon metabolism are significantly impacted by water stress, highlighting the plant’s metabolic plasticity. Under drought conditions, glycolytic metabolites surge, suggesting an energy conservation strategy. Conversely, flooding enhances Calvin cycle metabolites, indicating a photosynthetic adaptation. Notably, the resumption of regular watering prompts a swift metabolic rebound, underscoring the plant’s ability to restore normal physiological activities.

Collectively, these studies deepen our understanding of how plants reprogram their metabolism in response to environmental stresses and highlight the complexity of plant metabolic responses and the potential for manipulating these pathways to enhance stress tolerance. By revealing the metabolic underpinnings of plant resilience, these studies may open avenues for developing crops that can maintain productivity under challenging conditions.

## 4. Metabolomic Insights into Sorghum Seedling Development and Tomato Heat Stress Management

Sorghum, an important crop in arid regions, faces considerable challenges during its early developmental stages, particularly from soil-borne pathogens. Dubery et al. (contribution 9) provide a thorough examination of the metabolic reprogramming in sorghum seedlings, offering insights that are essential for enhancing crop resilience and ensuring food security, as it uncovers the role of specialized metabolites in plant growth, development, and defense mechanisms against environmental stresses, including the common damping-off disease in sorghum seedlings. Using targeted metabolomics, the research identified shifts in metabolite patterns across various growth stages, revealing a significant increase in metabolite content as the plants mature. Notably, the study found a correlation between apigenin flavone derivatives and growth stages, which could play a key role in the development of stress-resilient crops and the improvement of crop quality and yield.

Amidst the relentless rise in global temperatures, the agricultural sector faces significant challenges, particularly for heat-sensitive crops like tomatoes. Khan et al. (contribution 10) offer an exhaustive analysis of the adverse effects of heat stress on tomato plants. The authors detail how elevated temperatures negatively impact tomato growth, nutrient availability, photosynthesis, reproduction, and cellular integrity. Excessive production of reactive oxygen species (ROS) under heat stress is especially alarming, as it can lead to substantial cellular damage. The review emphasizes the need to develop heat-tolerant tomato varieties and highlights the potential of modern functional genomics techniques—transcriptomics, proteomics, and metabolomics—to identify key genes, proteins, and metabolites associated with heat stress tolerance. The integration of these technologies with traditional breeding methods could lead to the creation of robust tomato cultivars capable of withstanding high temperatures.

## 5. Conclusions

In conclusion, the comprehensive insights provided by the studies in this Special Issue underscore the pivotal role of metabolomics in plant defense mechanisms against both biotic and abiotic stresses. The observed metabolic reprogramming across diverse plant species, including tomatoes, barley, tea, bananas, and others, highlights the dynamic nature of plant responses to pathogens and environmental challenges. While these studies provide valuable insights into plant metabolic adaptations under stress, their focus on single stress factors or model crops might limit our understanding of combinatorial stress responses and their broader applicability in agricultural systems. To address these gaps and further explore the complexity of plant–environment interactions, we are organizing a follow-up Special Issue, “Metabolomics and Plant Defence, 2nd Edition”. This new issue will emphasize multi-omics integration, cross-species comparative analyses, and field-based studies under realistic climate conditions. The integration of metabolomics with other omics technologies and traditional breeding methods can facilitate the development of resilient, stress-adapted crop varieties, thereby contributing to the establishment of sustainable and climate-resilient agricultural systems.
